# Brainstem dominant form of X‐linked adrenoleukodystrophy with a novel ABCD1 missense variant: A case report and literature review

**DOI:** 10.1002/mgg3.2499

**Published:** 2024-07-25

**Authors:** Yulai Kang, Lu Guo, Zhuo Min, Lei Zhang, Lili Zhang, Chunhua Tang

**Affiliations:** ^1^ Department of Neurology and Centre for Clinical Neuroscience, Daping Hospital, Army Medical Centre of PLA Army Medical University Chongqing China

**Keywords:** ABCD1, brainstem, MRI, very long‐chain fatty acids, X‐linked adrenoleukodystrophy

## Abstract

**Background:**

X‐linked adrenoleukodystrophy (X‐ALD) is the most common peroxisomal disorder attributed to ABCD1 mutations. Case reports with predominant brainstem involvement are rare.

**Case Presentation:**

In this study, we reported a plateau male worker of X‐ALD characterized by progressive weakness accompanied by gait instability, mild nystagmus, and constipation. After 2 years of onset, a brain Magnetic Resonance Image (MRI) scan showed no abnormality but genetic analysis revealed a heterozygous mutation (c.1534G>A) in the ABCD1 gene. After 7 years of onset, although the patient was given aggressive dietary and symptomatic treatment in the course of the disease, a brain MRI scan showed predominantly brainstem damage, but serum concentrations of very long‐chain fatty acids were normal, and he had been bedridden for almost 2 years with severe bladder dysfunction, forcing him to undergo cystostomy. The patient was discharged with improved urinary retention and renal function.

**Conclusions:**

We reported an X‐ALD patient with a novel ABCD1 variation characterized by brainstem damage and retrospectively summarized the clinical manifestation, MRI features, and genetic features of X‐ALD patients with brainstem damage.

## 1 INTRODUCTION

X‐linked adrenoleukodystrophy (X‐ALD) is a common, progressive peroxisomal disorder. Due to different types of adenosine triphosphate‐binding cassette subfamily D, member 1 (ABCD1) gene variations, these hereditary factors lead to the deficiency of peroxisomal enzymes and the accumulation of very long‐chain fatty acids (VLCFAs) in the brain white matter and adrenal glands, resulting in central nervous system lesions and adrenal dysfunction (Gujral & Sethuram, 2023). X‐ALD is typically observed in male patients and exhibits a highly diverse and complex clinical spectrum (Köhler et al., 2018). This disease is mainly classified according to clinical manifestations and age of onset. Cerebral ALD is the most common type in children, and adrenomyeloneuropathy (AMN) is the most common type in adults (Zhu et al., 2020). Typical demyelinating lesions on the brain magnetic resonance image (MRI) support X‐ALD diagnosis, and elevated levels of VLCFAs and mutations in the ABCD1 gene can help confirm the diagnosis (Engelen et al., 2012). We report a case of an adult male with X‐ALD who was genetically diagnosed with a missense mutation (c.1534G>A) of the ABCD1 gene, with normal very long chain fatty acids and no abnormalities on MRI at 2 years of onset and with brainstem lesion at 7 years of onset as the main feature.

## 2 CASE REPORTS

### 2.1 Two hospitalizations

#### 2.1.1 January 2018

A 25‐year‐old male highland worker was admitted to the Neurology Department of Army Medical Center on 24 January 2018. The patient gradually developed lower limb weakness 2 years before admission, mainly in the right lower limb, which was obvious after activity. He could still walk independently and did not seek medical assistance. He realized that his fatigue had worsened, and he began to walk unsteadily, needing assistance to walk, with numbness and discomfort in both lower limbs and slight constipation 6 months before admission. During the disease, the strength of the upper limbs was normal without special discomfort. No choking or dysphagia occurred, and urination was normal. He reported slurbled rapid speech as a child. He denied other medical histories and had no history of exposure to drugs, Nitrous Oxide, or poison. The patient's parents denied a history of consanguineous marriage, family history, and similar clinical symptoms.

Physical examination: temperature: 36.7°C, heart rate: 78 beats/min, respiratory rate: 19 times/min, and blood pressure: 114/53 mmHg (1 mmHg = 0.133 kPa). No pigmentation is found in the skin mucosa; the hair is dark and thick. Neurological examination: clear consciousness and normal advanced cognitive function. Kayser‐Fleischer rings were not found in the corneas. Visual acuity and visual field were normal. An asymmetric gaze‐evoked nystagmus was detected. The muscle strength of the upper limbs was grade 5, and the muscle strength of the lower limbs was grade 5‐. Increased muscle tension of lower limbs, hyperreflexia of limbs, clonus of knees, and clonus of ankles were detected. Rotation test (−), Finger‐to‐nose test (−), Romberg sign (+). Deep sensation decreased in the lower limbs, but the superficial sensation was normal. Bilateral Hoffmann sign (+), Bilateral Rossolimo sign (+), Bilateral Babinski sign (+), Bilateral Chaddock sign (+). There was no resistance in the neck.

**FIGURE 1:**
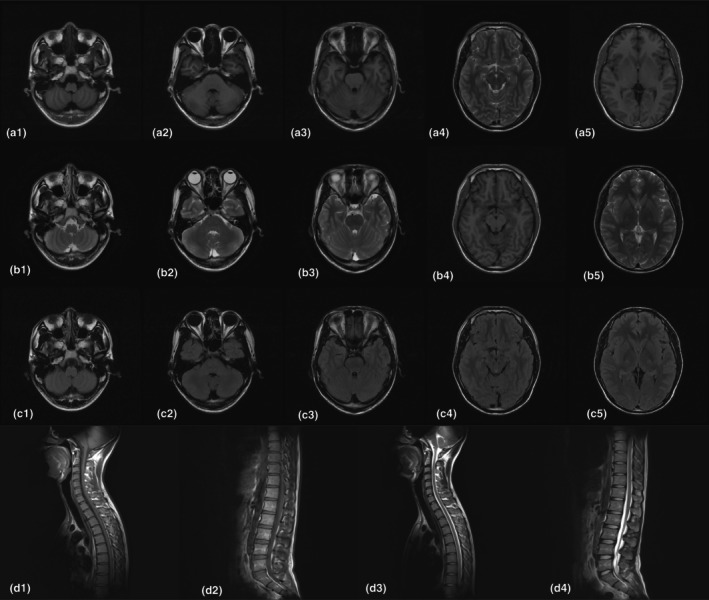
Magnetic resonance imaging (MRI). (a–c) Axial brain MRI scans showed no obvious abnormalities at five levels: cerebellum, pontocerebellar peduncle, brainstem, midbrain, and internal capsule. a = T1‐weighted MRI, b = T2‐weighted MRI, c = fluid‐attenuated inversion recovery. (d) Saggital spinal cord MRI scans showed no obvious abnormalities.

**FIGURE 2:**
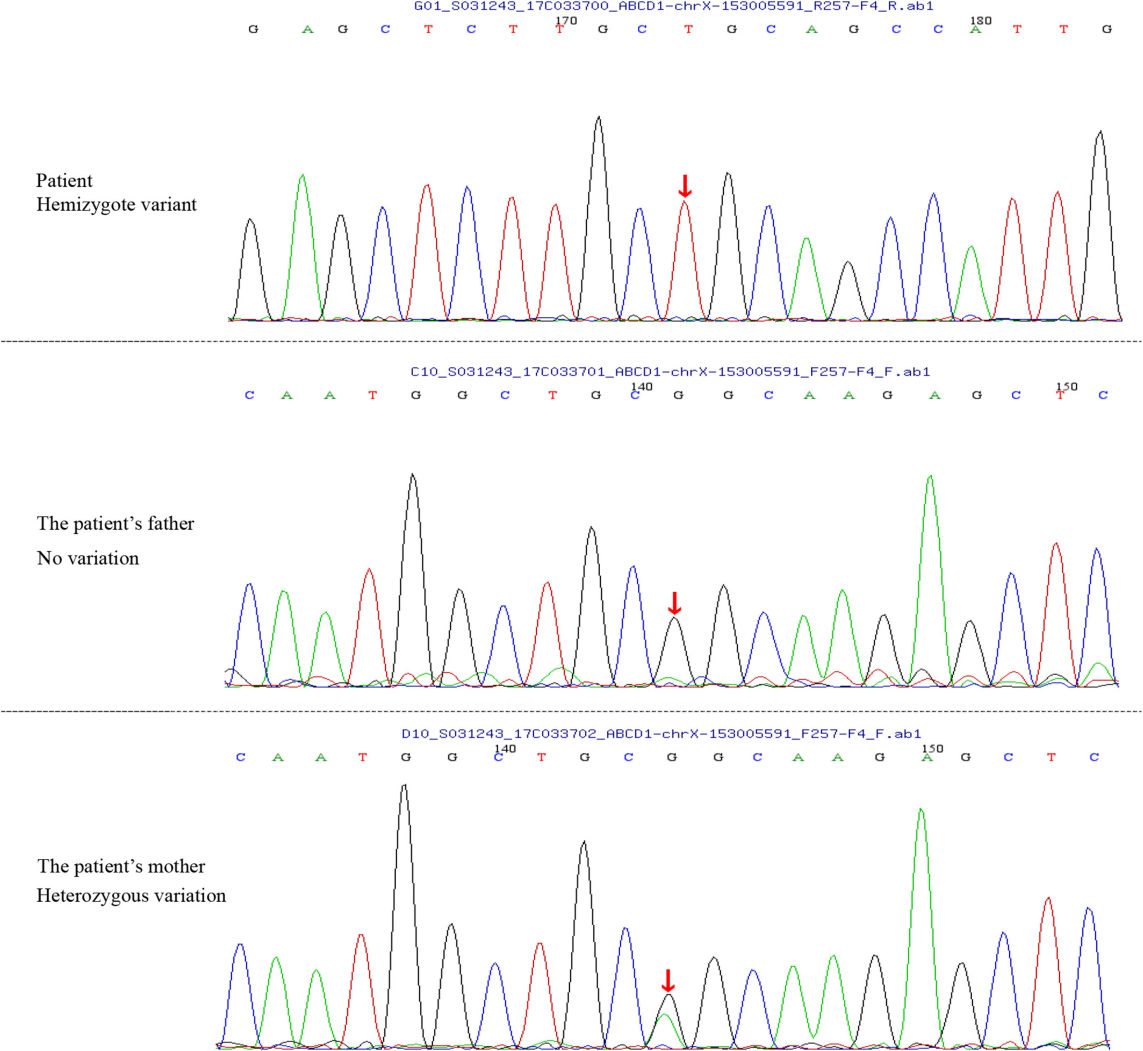
Adenosine triphosphate‐Binding Cassette subfamily D, member 1 (ABCD1) gene sequencing of the X‐linked adrenoleukodystrophy patient and his parents. The arrow showed the patient with a missense mutation (c.1534G>A) in the ABCD1 gene, his mother with heterozygous variation at the same spot, and his father with no variation.

Examinations after admission: Serum: Blood routine, liver and kidney function, thyroid function, rheumatic immunity, folic acid, vitamin B12, ferritin, coagulation test, tumor markers, neural antigen–antibody spectrum, pretransfusion pathogens were normal. Adreno‐corticotrophic hormone (ACTH): 11.50 pg/mL (range 5.08–32.86); cortisol: 275 ng/mL (range 50–280). Mass spectrometry of the blood and urine was negative. Figure 1 shows that brain and spinal cord MRI revealed no abnormalities. Electromyography/evoked potential examination: (1) BAEP abnormalities; (2) P300 wave: normal; (3) VEP: normal; (4) Median nerve: SEP normal; (5) Posterior tibial nerve: SEP severely abnormal; (6) Median nerve, ulnar nerve, radial nerve: SCV normal; (7) Median nerve and ulnar nerve: MCV normal; (8) Posterior tibial nerve and common peroneal nerve: MCV normal; (9) Tibial nerves and peroneal nerve: SCV abnormal; (10) Tibial nerve and peroneal nerve: MCV normal; (11) Right tibial nerve: H‐reflex abnormality and F wave abnormal; (12) Left gastrocnemius muscle: EMG showed neurogenic changes. ABCD1 gene sequencing was performed on the patient and his parents with the informed consent of the patient and his family. The patient was found to have a hemizygous variant (c.1534G>A) at the chrX‐153005591 locus in the ABCD1 gene, which was a missense mutation and was considered to be likely pathogenic and associated with adrenoleukodystrophy based on the guideline of the American College of Medical Genetics and Genomics (Figure 2).

Combined with clinical manifestations and gene sequencing results, the patient was finally diagnosed with X‐ALD. Levodopa and benserazide, mecobalamin tablets, and eperisone were given symptomatic treatment. He was also instructed to reduce the intake of a VLCFAs‐rich diet and had been taking Lorenzo's soil intermittently, but no significant improvement in symptoms was observed. Due to factors such as economic status and the COVID‐19 epidemic, the patient did not seek further treatment after discharge until July 2023.

#### 2.1.2 July 2023

The patient was readmitted on 4 July 2023. In fact, since 2021, the patient has been in bed for a long time and gradually developed urination disorders, defecating every 4–5 days, and a month ago, the patient began to develop right upper limb weakness and dysarthria. He denied any history of COVID‐19 vaccination.

**FIGURE 3:**
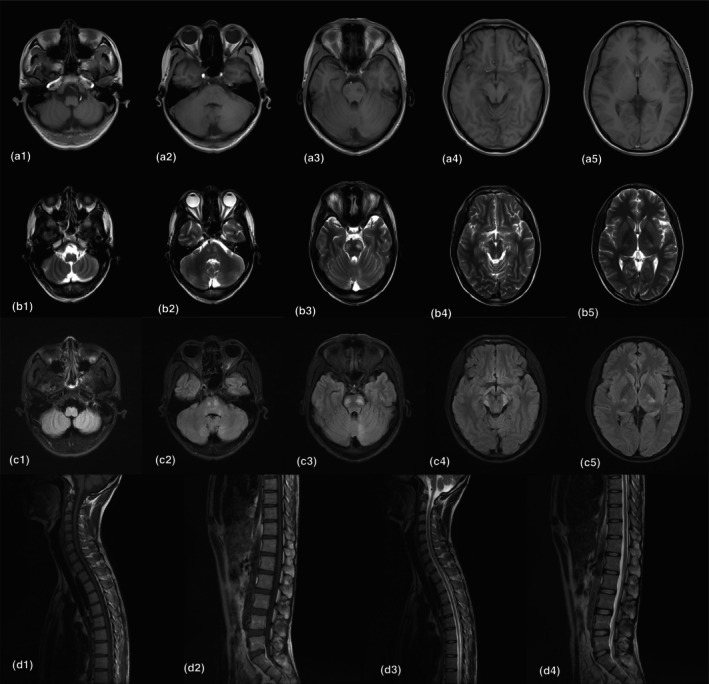
Magnetic resonance imaging (MRI). (a–c) Axial brain MRI scans showed distribution of lesions at five levels: cerebellum, cerebellopontine angle, brainstem, midbrain, and internal capsule. a = T1‐weighted MRI, b = T2‐weighted MRI, c = fluid‐attenuated inversion recovery. (d) Saggital spinal cord MRI scans showed thoracic spinal cord atrophy.

Changes in examination since the last admission: Wheelchair admission, speech is not fluent, both upper limbs biceps, triceps reflex, and radial reflex are abnormally active. The muscle tone of the right upper limb increased in a knife‐like manner, the muscle strength of the right upper limb was grade 5‐, and the muscle strength of the lower limbs was grade 2. Copper ion and ceruloplasmin were normal; the ACTH level was 86.88 pg/mL (normal range 5.08–32.86). The estimated glomerular filtration rate was 21.04 mL/min/1.73m^2^. The level of VLCFAs is shown in Table 1: C26: 0.525 nmol/L (range 0.17–1.73), C24/C22 ratio: 0.839 (normal levels 0.64–1.02), and C26/C22 ratio: 0.0128 (normal levels 0.002–0.015). Ultrasound showed abnormal kidney morphology with severe hydrops. Spinal cord MRI showed thoracic spinal cord atrophy. Brain MRI showed symmetrical distribution of abnormal signal shadow in the bilateral optic tract, basal ganglia, brainstem, and cerebellar hemisphere, showing long T1 T2 patchy signal shadow. FLAIR showed hyperintensity. (Figure 3).

**TABLE 1:** Very long chain and branched chain fatty acid test report.

Inspection item	Value	Reference range	Unit
C22:0	41.0	28.94–93.50	μmol/L
C24:0	34.4	24.25–77.75	μmol/L
C26:0	0.525	0.17–0.73	μmol/L
C26:0/C22:0	0.0128	0.003–0.015	
C24:0/C22:0	0.839	0.64–1.02	
C20:0	0.290	0.25–2.07	μmol/L
C19:0	0.078	<0.26	μmol/L
C19:0/ C20:0	0.268	<0.28	

During the diagnosis and treatment, in addition to the above treatments, the patient was given drugs to improve renal function and then transferred to the urology department for a cystostomy. The patient was discharged with improved urinary retention and renal function.

## 3 DISCUSSION

X‐ALD is a recessive genetic disorder caused by a mutation of the ABCD1 gene on the X chromosome (Gujral & Sethuram, 2023). The most common type among children is childhood cerebral ALD, whereas AMN is the most prevalent among adults. Addison‐only is also one phenotype of X‐ALD, the primary clinical feature is primary adrenal insufficiency, with no involvement of nervous system. The onset typically occurs before 7.5 years old and ultimately progresses to AMN. X‐ALD, characterized by brainstem and/or cerebellar signs and symptoms, is a rare phenotype, accounting for 1%–2% (Kemp et al., 2001). Past studies have taken different nomenclatures for such patients who are statistically more common in East Asia. Apart from the differences in MRI manifestations, patients with brainstem type often present with dysarthria and motor dysfunctions. Compared with other phenotypes, cognitive disorder are less common.

**FIGURE 4:**
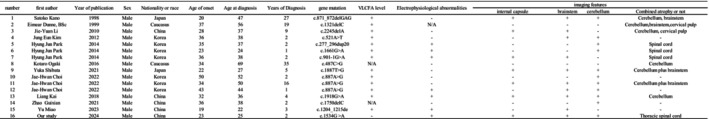
Baseline features, electrophysiological results, imaging features, and gene variation of 16 patients with X‐linked adrenoleukodystrophy characterized by brainstem symptoms.

**FIGURE 5:**
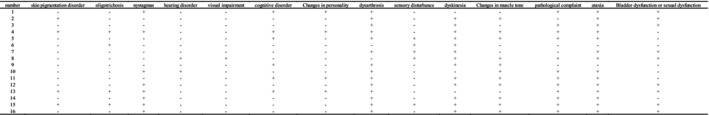
Clinical characteristics of 16 patients with X‐linked adrenoleukodystrophy characterized by brainstem symptoms.

Currently, the diagnostic methods of X‐ALD include clinical symptoms and signs, imaging examination, plasma VLCFAs levels, and ABCD1 gene sequencing (Engelen et al., 2012; Gujral & Sethuram, 2023). After the literature review, with “adrenoleukodystrophy” and “brainstem” as keywords, we searched PubMed, Excerpta Medica Database, Cochrane Library, China National Knowledge Infrastructure, and WanFang database from 1980 to the present. Combined with our reported patients, 16 cases of X‐ALD with predominantly brainstem damage were confirmed by gene sequencing (Choi et al., 2022; Dunne et al., 1999; Kano et al., 1998; Kim et al., 2012; Li et al., 2010; Liang et al., 2018; Ogaki et al., 2016; Park et al., 2014; Shibata et al., 2021; Yu & Yue, 2023; Zhao, 2021). We summarized the characteristics of these patients with brainstem involvement in detail (Figures 4 and 5). All patients are male. 87.5% (14/16) of the patients were from East Asia. The average age of onset was 31.75 ± 8.66 years. The average age at diagnosis was 40 ± 12.72 years. The average time from onset to diagnosis was 8 years, with the shortest diagnosis being 1 year and the longest being 35 years. From the perspective of mutation type, nine patients (56.25%) had missense mutations, five patients (31.25%) had deletion mutations, and 2 patients (12.5%) had frameshift mutations. All VLCFAs levels were abnormal except in the patient we reported. 46.6% of the patients had abnormal electrophysiological examination results. Dysarthria (87.5%), ataxia (87.5%), and Dyskinesia (81.3%) were the most common clinical manifestations. Pathological complaint (75%), changes in muscle tone (68.8%), bladder dysfunction or sexual dysfunction (56.25%), and Nystagmus (50%) were common clinical manifestations. Other possible clinical symptoms and signs may include: skin pigmentation disorder, oligotrichosis, cognitive disorder, Changes in personality, and sensory disturbance. Hearing disorders and visual impairment were rare. MRI features showed that 62.5% of patients had abnormal T2 signaling in the brainstem, and 68.75% had atrophy in the brainstem, cerebellum, or spinal cord.

The present case is a young man. Thanks to the patience and care of the first physician, the patient received a precise diagnosis by gene sequencing at the first medical visit and corresponding dietary and symptomatic treatment. He survived longer and completed a second visit, and the appropriate follow‐up is still ongoing via telephone. In contrast to other case reports, we report novel missense mutations (c.1534G>A) that lead to brainstem damage, although this variant has been reported in other types of X‐ALD (Kok et al., 1995). It is worth noting that during the second hospitalization, the levels of VLCFAs were normal. This could possibly be attributed to an early diagnosis, allowing the patient to undergo long‐term dietary therapy, which managed to metabolize the VLCFAs back to normal levels (Zhu et al., 2020). In this case, due to the limitations of the patient's age and the negative results of the initial MRI examination, Hematopoietic stem cell transplantation treatment was not pursued. Instead, dietary therapy was chosen, which effectively delayed the progression of the disease but did not achieve reversal (Gujral & Sethuram, 2023; Köhler et al., 2018). We noted that the patient's ACTH levels and MRI showed abnormalities in the seventh year. Regarding the atrophy site, spinal cord thoracic segment atrophy is equally rare compared with other case reports. During follow‐up, we remained committed to improving the patient's quality of life by performing a cystostomy, even though he was forced to be bedridden.

## 4 CONCLUSION

This case study contributes to the understanding of X‐ALD with primarily brainstem damage. When encountering young adult males with typical neurological symptoms, particularly those with a short duration of illness and normal results from biochemical and imaging examinations, a more thorough investigation should be conducted to facilitate timely recognition, diagnosis, and early intervention.

## AUTHOR CONTRIBUTIONS

Yulai Kang and Lu Guo: Data curation, writing—original draft. Zhang Lei and Zhuo Min: Writing—review & editing, supervision. Lili Zhang and Chunhua Tang: Conceptualization, writing—review & editing, supervision. All authors had read and approved the final version of the manuscript.

## FUNDING INFORMATION

This work was supported by the Natural Science Foundation Project of CQ CSTC (cstc2019jscx‐msxmX0285) and the Natural Science Foundation Project of Chongqing (CSTB2022NSCQ‐MSX1585).

## CONFLICT OF INTEREST STATEMENT

The authors have no competing interests to declare.

## ETHICS STATEMENT

The ethics committee of the Army Medical Centre of PLA approved this study.

## CONSENT

Written informed consent was obtained from patient.

## Data Availability

The data that support the findings of this study are available from the corresponding author, [Chunhua Tang], upon reasonable request.
